# TWIST and p-Akt immunoexpression in normal oral epithelium oral dysplasia and in oral squamous cell carcinoma

**DOI:** 10.4317/medoral.17344

**Published:** 2011-07-15

**Authors:** Brunno-Santos de Freitas Silva, Fernanda-Paula Yamamoto, Flávia-Sirotheau Corrêa Pontes, Sérgio-Elias Cury, Felipe-Paiva Fonseca, Hélder Rebelo-Pontes, Décio-dos Santos Pinto-Júnior

**Affiliations:** 1 DDS, MSc - PhD student, Department of Oral Pathology, Dental School, University of São Paulo, São Paulo, São Paulo, Brazil; 2 DDS - PhD student, Department of Oral Pathology, Dental School, University of São Paulo, São Paulo, São Paulo, Brazil; 3 DDS, MSc, PhD – Department of Oral Pathology, Dental School, Federal University of Pará, Belém, Pará, Brazil; 4 DDS, PhD - Department of Stomatology, Dental School, UNIFOA, Volta Redonda, Rio de Janeiro, Brazil; 5 DDS – MSc student, Department of Oral Pathology, Dental School, Piracicaba-UNICAMP, Piracicaba, São Paulo, Brazil; 6 DDS, MSc, PhD - Department of Oral Pathology, Dental School, University of Sao Paulo, São Paulo, SP, Brazil

## Abstract

Objectives: The aim of this study was to evaluate the immunoexpression of TWIST and p-Akt proteins in oral leukoplakia (OL) and oral squamous cell carcinoma (OSCC), correlating their expressions with the histological features of the lesions. Study design: Immunohistochemical studies were carried out on 10 normal oral epithelium, 30 OL and 20 OSCC formalin-fixed, paraffin-embedded tissue samples. Immunoperoxidase reactions for TWIST and p-Akt proteins were applied on the specimens and the positivity of the reactions was calculated for 1000 epithelial cells. Results: Kruskal-Wallis and Dunn’s post tests revealed a significant difference in TWIST and p-Akt immunoexpression among normal oral mucosa, OL and OSCC. In addition, a significant positive correlation was found between TWIST and p-Akt expressions according to the Pearson’s correlation test. Conclusions: The results obtained in the current study suggest that TWIST and p-Akt may participate of the multi-step process of oral carcinogenesis since its early stages.

** Key words:** Oral cancer, oral leukoplakia, dysplasia, immunohistochemistry.

## Introduction

Oral squamous cell carcinoma (OSCC) is the most prevalent malignancy of the oral cavity usually associated with a poor prognosis (1). Most cases of OSCC are preceded by visible changes of the oral mucosa (2). Therefore, it would be important to identify those changes that may represent an early stage of the process of malignant transformation (3). Oral leukoplakia (OL) is considered the most prevalent potentially malignant lesion of the oral mucosa (4,5). This term should be used to recognize white plaques of questionable risk having excluded (other) known diseases or disorders that carry no increased risk for cancer (4). The presence of epithelial dysplasia seems to be an important prognostic indicator of malignant transformation (3). However, accuracy of dysplasia grading is dependent on the quality of the specimen and the location of the lesion where the biopsy was done. In addition, this grading system is also too subjective, with inter and intra-observers variability (6,7). Therefore, studies evaluating the effectiveness of molecular markers in predicting the prognosis of premalignant lesions are relevant (8).

Recent studies evaluated the role of different molecular markers as adjuvants in identifying the malignant potential of potentially malignant oral lesions presenting different degrees of epithelial dysplasia (9-11). Hence, p-Akt (protein kinase B) protein has been the target of different studies (9,10) and it has been considered an important oncogene responsible for the development of a wide variety of malignancies (9,12), including OSCC (9).

TWIST is a basic helix-loop-helix highly conserved transcription factor that plays a key role in the progression of a primary tumor to the metastatic stage (13). It has been reported that TWIST may serve as an independent prognostic marker for predicting distant metastasis and the survival rate of patients affected by esophageal squamous cell carcinoma (14). TWIST also seems to play an important role in OSCC progression and lymph node metastasis (15). A recent study showed that p-Akt is a transcriptional regulatory target of TWIST in breast cancer cells, and its activation resulted in cell survival, migration and invasion (16).

The aim of this study was to evaluate the immunoexpression of TWIST and p-Akt proteins in OL and OSCC, with the purpose of verifying the participation of these proteins in the malignant transformation of oral epithelium.

## Material and Methods

 -Specimens 

Thirty paraffin-embedded tissue samples of OL (Mild dysplasia n=10, moderate dysplasia n=10 and severe dysplasia n=10), 20 of OSCC and 10 of normal oral mucosa obtained from the floor of the mouth, tongue, palate and gingival of 41 males and 19 females, all smokers, with a mean age of 47.6 year-old, were selected from the surgical pathology archives of the Department of Oral Pathology of the University of São Paulo. The samples were submitted to 5μm histological sections, to routinely staining with haematoxylin and eosin (H&E;) and analyzed under light microscopy. The histological grades of oral dysplasia were determined by two independent oral pathologists according to the World Health Organization (WHO) criteria (17) in a blinded fashion. Any discrepancies in the findings were analyzed and discussed among the observers for a final evaluation. 

 -Immunohistochemical staining and evaluation

Three-µm 4% formalin-fixed slides were dewaxed with xylene and hydrated in ethanol series. For antigen retrieval, sections that received the antibody anti-TWIST were immersed in 10 mM monohydrated citrate buffer solution (pH 6.0) and heated in microwave oven at 95°C for 15 minutes. Slides that received the antibody anti-p-Akt 1 ⁄ 2 ⁄ 3 were immersed in 10 mM monohydrated citrate buffer solution (pH 6.0), in water-bath at 95°C for 30 minutes. Peroxidase activity was blocked with 6% hydrogen peroxide and methanol solution in two baths of 15 minutes each at room temperature. After washing with Tris buffer (pH 7.4), the slides were incubated with the primary antibodies anti-TWIST (H-81 – Sc:15396; Santa Cruz Biotechnology, CA, USA), dilution 1:100, incubated at 4°C overnight, and anti-p-Akt 1 ⁄ 2 ⁄ 3 (Thr 308 – Sc:16646-R; Santa Cruz Biotechnology, Santa Cruz, CA, USA), dilution 1:100, incubated at room temperature for 60 minutes. The slides were subsequently exposed to the avidin-biotin complex (LSAB-Kit + HRP; Dako Cytomation, Carpinteria, CA, USA) and to the 3,3’-diaminobenzidin chromogen (DAB+; Dako Cytomation, Carpinteria, CA, USA). The sections were counterstained with Meyer haematoxylin, dehydrated in ethanol, cleared in xylene and mounted. Breast cancer tissues were used as positive control for TWIST and prostate adenocarcinoma sections for p-Akt protein. The negative control was obtained by omitting the primary specific antibody during the reaction. The immunohistochemical sections were analyzed by one independent blind and calibrated observer under light microscopy at 400x magnification. Five histological fields were randomly chosen and 1000 cells were counted in each slide. The analysis was carried out taking in count the number of positive cells in cytoplasmatic and/or nuclear compartments.

 -Statistical analysis

The statistical analysis was performed using BioEstat 5.0 software (Mamirauá Institute, Belém, PA, Brazil). Possible differences in TWIST and p-Akt immunoexpression among the five groups studied were verified by non-parametric variance method, the Kruskal-Wallis test. Dunn’s post test was applied to determine which groups significantly differed from each other in respect to the means. Pearson’s correlation test was used to determine the possible existence of correlation between TWIST and p-Akt expression. The p-value < 0.05 was considered to be statistically significant. This study was approved by the ethical committee of the University of São Paulo. 

## Results

**Figure 1:**
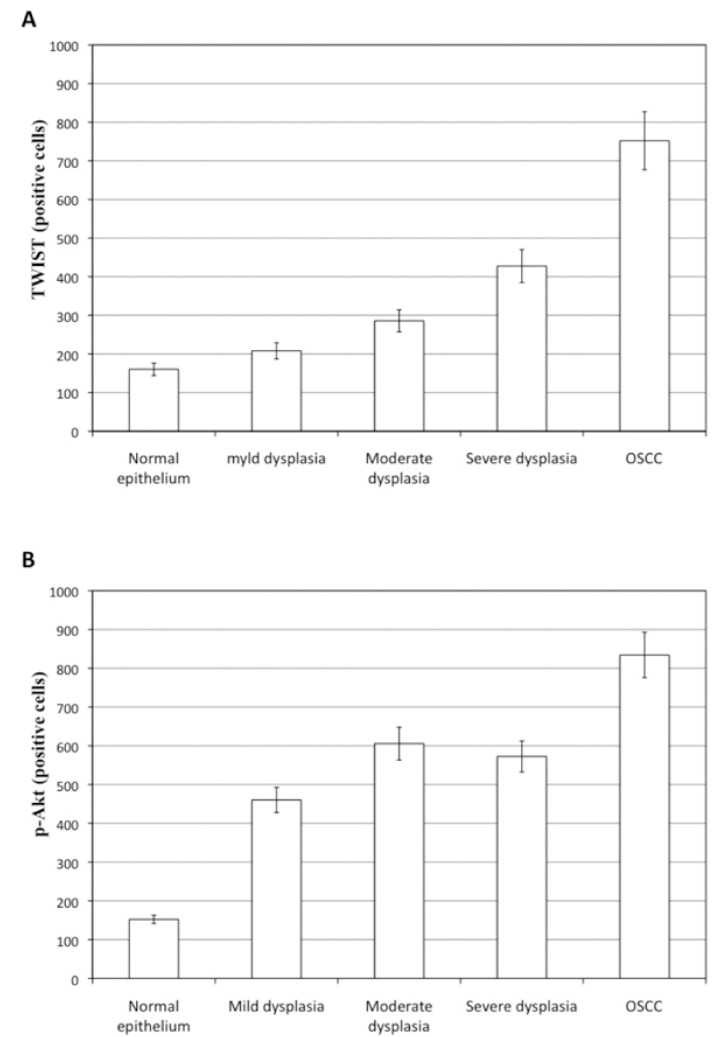
A) TWIST expression (positive cells) among the normal oral mucosa, OL and OSCC groups. B) p-Akt expression (positive cells) among the normal oral mucosa, OL and OSCC groups.

**Figure 2:**
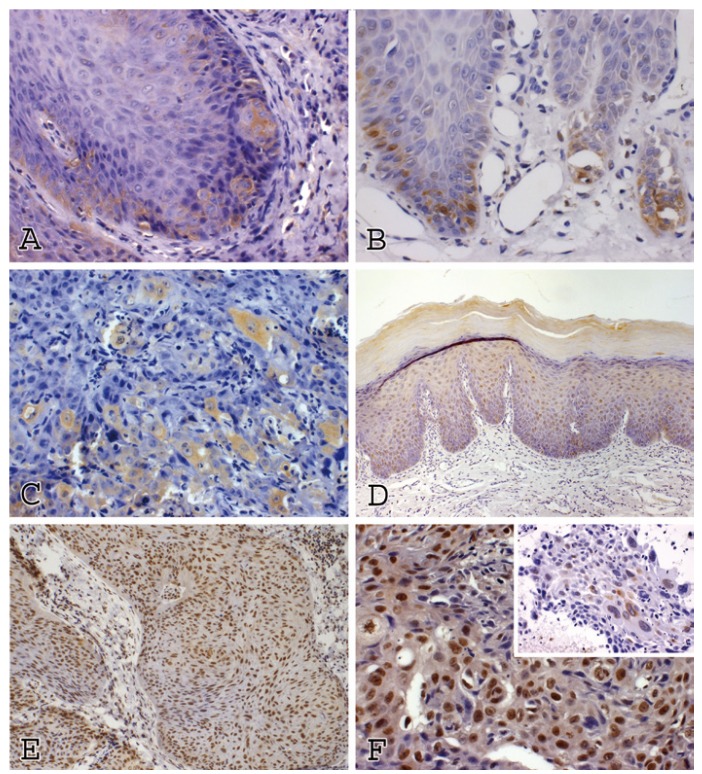
Immunoexpression of TWIST and p-Akt proteins (streptavidine-biotine). A) TWIST expression in oral epithelium with moderate dysplasia (x400). B) TWIST expression in oral epithelium with severe dysplasia (x400). C) TWIST expression in OSCC (x200). D) p-Akt expression in oral epithelium with moderate dysplasia (x200). E) Oral epithelium with severe dysplasia (x200). F) p-Akt expression in OSCC (x400).

Kruskal-Wallis test showed a statistically significant variance in TWIST immunoexpression among the five groups (P< 0.01). Dunn’s post test pointed significant differences in TWIST immunoexpression between normal oral mucosa and OSCC (P= 0.0001), mild dysplasia and OSCC (P= 0.0022), moderate dysplasia and OSCC (P= 0.0075), and between severe dysplasia and OSCC (P= 0.0071). It was also observed differences in TWIST expression between moderate dysplasia and severe dysplasia (P= 0.0475). However, no significant differences were observed between normal oral mucosa and mild dysplasia, and between mild dysplasia and moderate dysplasia (Fig. 1). TWIST immunoreaction in normal oral mucosa and in all OL groups (mild, moderate and severe dysplasia) was predominantly found in the parabasal and basal layers, showing a cytoplasmatic staining. In OSCC, TWIST showed a diffuse distribution in neoplastic islands, with a higher expression in poorly differentiated tumors, mainly revealing a citoplasmatic localization (Fig. 2); however, nuclear staining was sometimes also observed.

p-Akt immunoexpression, according to the Kruskal-Wallis test, showed a significant difference among the five groups (P= 0.0083). Dunn’s post test pointed significant differences in p-Akt immunoexpression in normal oral mucosa if compared to the three OL groups (mild, moderate and severe epithelial dysplasia) (P< 0.01), and between the three OL groups and OSCC (p< 0.05). There was no statistical difference among the three OL groups (Fig. 1). 

In the normal oral mucosa, p-Akt staining was observed to be restricted to the nucleus and limited to the basal and parabasal cells. In OL groups, p-Akt was also restricted to the nucleus, but it was present in almost all epithelial layers. Finally, in OSCC group, p-Akt immunoexpression was found in both cellular compartments, with diffuse localization in both central and peripheral areas of tumor islands (Fig. 2). 

**Figure 3:**
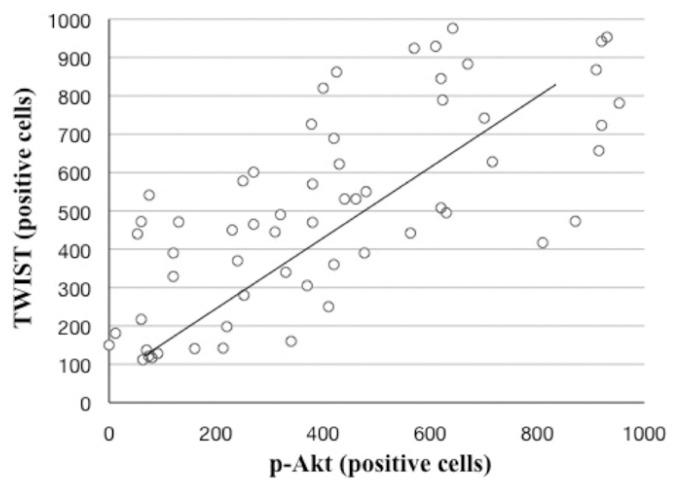
Scatter plot illustrating the positive association between TWIST and p-Akt expression under Pearson’s correlation test.

There was a significant positive correlation between TWIST and p-Akt immunoexpression in normal oral mucosa, OL and OSCC (Pearson’s correlation test, r= 0.8741, P= 0.0426) (Fig. 3).

## Discussion

The development of OSCC is associated with genetic changes that result in absence of controlling mechanisms related to cell growth and differentiation (2). Studies have shown that these genetic alterations are also present in premalignant lesions, suggesting a possible role in the malignant transformation process (9,18). The present study showed significant differences in TWIST and p-Akt proteins expression among normal oral mucosa, OL and OSCC, suggesting that these two proteins may participate in the multi-step process of oral carcinogenesis. To our knowledge, this is the first study to evaluate TWIST expression in OL. Interestingly, TWIST immunoreaction in all OL groups was predominantly found in the parabasal and basal layers, where these regions are known to initiate the dysplastic changes in OL (19).

Progression of OSCC may be associated with loss of E-cadherin, resulting in a more invasive phenotype (20). TWIST acts in EMT as a repressor of E-cadherin, promoting the loss of cell-to-cell adhesion and causing a dramatic remodeling of cytoskeleton, increasing cell motility (21). This may explain the phenotypic change found in TWIST positive cells in our study.

TWIST, a highly conserved basic helix-loop-helix transcription factor, that was found to be a key factor in the promotion of metastasis of cancer cells, is known to induce epithelial-mesenchymal transition (EMT), a recently identified event implicated in tumor metastasis, and to promote cancer cell invasion, both important steps in cancer progression (22). TWIST participation in cancer progression and metastasis has been reported in a variety of tumors, including breast cancer (16), prostatic cancer (22), pancreatic cancers (23), gastric cancer (21), cervical cancer (24), bladder cancer (13,25) and OSCC (15,26). However, little is known about its involvement in the progression of OSCC.

TWIST immunoexpression was found in the cytoplasm and nucleus of the head and neck squamous cell carcinoma specimens, but no assumption was made about these findings (15). In our study, TWIST was mainly found in the cytoplasm of normal oral mucosa and OL. In OSCC TWIST protein was mostly observed in the cytoplasm; however, a nuclear staining was also observed in a few cases, similar to the reported by Ou et al. (15). In OSCC group, TWIST expression was related with differentiation status, with a higher immunoexpression in poorly differentiated tumors.

According to Yuen et al. (22), increased cytoplasmatic immunostaining of TWIST is positively associated with neoplastic transformation in prostatic tissue. This increased cytoplasmatic expression might play a negative role in the regulation of prostatic cancer cell differentiation, whereas increased nuclear expression of TWIST may play a positive role in promoting metastasis. Similar findings were found in the current study, supporting the possible participation of TWIST in OSCC progression and its possible value as a prognostic marker. 

TWIST seems to be a pivotal transcription factor that may positively regulate the expression of CXCR4 and CCR7 genes during lymph node metastasis, and its expression is significantly correlated with the clinical stage of head and neck squamous cell carcinoma (15). It has been reported that CXCR4 can be up-regulated through NF-kB in prostate and breast carcinomas (15, 27). Additionally, PI3K/Akt pathway activation seems to induce EMT via CXCR4 (28). Strong evidence shows that PI3K/Akt is required for activation of NFκB induced by TNF and interleukin 1 (IL-1), or through Ras protein with the activation of IKKβ (29). 

Recent studies suggest that TWIST is involved with p-Akt pathways (26,30). Both TWIST and p-Akt were associated with the downregulation of E-cadherin and the loss of cell-to-cell adhesion in EMT (22,26). Increased TWIST expression may influence p-Akt pathway through unclear mechanisms in nasopharyngeal carcinoma cells. However, this cross-talk may be explained by the possible interference of TWIST and p-Akt in the p53/MDM-2 pathway (30). Other study showed that p-Akt inhibition could induce the mesenchymal-to-epithelial reverting transition through interaction with NF-kB and downregulation of TWIST in OSCC cells (26), suggesting that there may be a positive interaction between TWIST and p-Akt during the EMT in OSCC. 

The serine/threonine protein kinase Akt is a downstream effector of phosphatidylinositol 3-kinase (PI3K) and is frequently activated in human cancers (26). p-Akt was found to be an independent prognostic factor in patients with OSCC (31), and has recently been linked with the conversion of a potentially malignant oral lesion to OSCC (9). Pontes et al. (9) demonstrated that p-Akt expression in OSCC in significantly increased when compared to epithelial dysplasia and normal epithelium, suggesting that p-Akt expression can be an early event in cases of dysplastic lesions progressing to oral cancer. These results are in accor-dance to the obtained in the present study, where p-Akt immunoexpression was significantly increased in OSCC if compared with normal oral mucosa and OL. 

In the current evaluation, p-Akt immunoexpression was found in the nucleus and cytoplasm. In normal oral mucosa and OL, p-Akt staining was restricted to the nucleus, whereas in OSCC p-Akt immunoexpression was found in both compartments, with a predominance of cytoplasmatic immunoreactivity in the less differentiated tumors. According to Amornphimoltham et al. (31) p-Akt can be detected in head and neck carcinogenesis with a pattern of expression and localization that correlates with the progression of the malignant process (normal epithelium, epithelial dysplasia, carcinoma in situ and OSCC). Indeed, they found that in normal epithelium the staining was limited to the basal and parabasal cell layers and always confined to the nucleus. In dysplastic areas they reported that the cells presented a strong nuclear staining, whereas in situ carcinoma revealed nuclear and cytoplasmatic staining. However, only in a few cases of invasive OSCC, p-Akt staining was nuclear; in most instances it was predominantly cytoplasmatic.

TWIST expression in parabasal and basal layers of OL groups associated with acquisition of an dysplastic phenotype at this region and the positive correlation between TWIST and p-Akt expression, found in this study, suggests that it may participate of oral carcinogenesis since early stages. However, the exact mechanism of this interaction requires further investigation. 

In conclusion, the results obtained in the current study suggest that TWIST and p-Akt may participate in the process of malignant transformation of oral epithelium since early stages of oral carcinogenesis process. Additionally, the high expression of TWIST and p-Akt in OSCC suggests that these proteins might be important targets for therapeutic approaches in patients affected by OSCC.
